# MicroRNAs Regulating Cytoskeleton Dynamics, Endocytosis, and Cell Motility—A Link Between Neurodegeneration and Cancer?

**DOI:** 10.3389/fneur.2020.549006

**Published:** 2020-11-09

**Authors:** Dmytro Gerasymchuk, Anastasiia Hubiernatorova, Andrii Domanskyi

**Affiliations:** ^1^Institute of Biotechnology, Helsinki Institute of Life Science (HiLIFE), University of Helsinki, Helsinki, Finland; ^2^Institute of Molecular Biology and Genetics, National Academy of Sciences of Ukraine, Kyiv, Ukraine

**Keywords:** microRNA, clathrin-mediated endocytosis (CME), neurodegenerative diseases, post-transcriptional regulation, cancer, actin cytoskeleton, cell motility

## Abstract

The cytoskeleton is one of the most mobile and complex cell structures. It is involved in cellular transport, cell division, cell shape formation and adaptation in response to extra- and intracellular stimuli, endo- and exocytosis, migration, and invasion. These processes are crucial for normal cellular physiology and are affected in several pathological processes, including neurodegenerative diseases, and cancer. Some proteins, participating in clathrin-mediated endocytosis (CME), play an important role in actin cytoskeleton reorganization, and formation of invadopodia in cancer cells and are also deregulated in neurodegenerative disorders. However, there is still limited information about the factors contributing to the regulation of their expression. MicroRNAs are potent negative regulators of gene expression mediating crosstalk between different cellular pathways in cellular homeostasis and stress responses. These molecules regulate numerous genes involved in neuronal differentiation, plasticity, and degeneration. Growing evidence suggests the role of microRNAs in the regulation of endocytosis, cell motility, and invasiveness. By modulating the levels of such microRNAs, it may be possible to interfere with CME or other processes to normalize their function. In malignancy, the role of microRNAs is undoubtful, and therefore changing their levels can attenuate the carcinogenic process. Here we review the current advances in our understanding of microRNAs regulating actin cytoskeleton dynamics, CME and cell motility with a special focus on neurodegenerative diseases, and cancer. We investigate whether current literature provides an evidence that microRNA-mediated regulation of essential cellular processes, such as CME and cell motility, is conserved in neurons, and cancer cells. We argue that more research effort should be addressed to study the neuron-specific functions on microRNAs. Disease-associated microRNAs affecting essential cellular processes deserve special attention both from the view of fundamental science and as future neurorestorative or anti-cancer therapies.

## Introduction

Since the discovery of microRNA in 1993 (1), these 18–25 nt non-coding molecules have been recognized as important post-transcriptional regulators of physiological and pathological processes in different tissues and organs. MicroRNA biogenesis is mediated by ribonucleases Drosha and Dicer in complex with several regulatory proteins controlling specificity and activity of these enzymes [reviewed in Bartel (2)] and is affected by multiple signal transduction and stress response pathways (3–8). Conversely, microRNAs mediate crosstalk between different cellular pathways forming large-scale regulatory networks that regulate cellular homeostasis and stress responses (2, 8–10). Therefore, it is not surprising that microRNAs are implicated in multiple pathological conditions. Indeed, microRNA levels change in aging, cancer, and neurodegenerative diseases [reviewed in Emde and Hornstein (5), Eacker et al. (11), Juzwik et al. (12), and Konovalova et al. (13)]. The relative ease of detection prompted research to exploit the possibility of using microRNAs as biomarkers in multiple diseases and conditions. The overwhelming majority of current clinical trials related to microRNAs are investigating the possibility to use circulating and exosomal microRNAs for early disease diagnosis (14–17). The application of microRNAs as therapeutic molecules has proven to be more challenging, but, nevertheless, a promising approach, and indeed modulation of particular microRNAs has been suggested for the therapy of cancers [reviewed in Hosseinahli et al. (18) and Hanna et al. (19)] and neurodegenerative diseases [reviewed in Sonntag (20), Junn and Mouradian (21), Sonntag et al. (22), and Wen (23)].

To date, the role of microRNAs in the development and disorders of the nervous system is investigated intensively (11). MicroRNAs are known to influence numerous genes involved in neuronal differentiation, plasticity and degeneration. For instance, in Alzheimer's disease (AD) several microRNAs can target key genes involved in the production of amyloid-beta (Aβ), inflammation and defects of neurotransmission: miR-124 targets Aβ-cleaving enzyme 1 BACE1 thus preventing Aβ formation, while downregulation of miR-15a promotes tau hyperphosphorylation (24, 25). MicroRNAs and their biogenesis machinery are also important disease-associated factors in Parkinson's disease (PD) (26, 27). Studies on post-mortem laser-microdissected dopaminergic neurons demonstrate a reduction of *DICER1* gene expression in PD patients (28). Dicer is essential for the function and survival of adult dopaminergic neurons and pharmacological stimulation of microRNA biogenesis pathway promotes dopaminergic neuron survival and protects them from cellular stress (29). The level of Dicer expression correlates with the severity of PD-related symptoms in mice, and several microRNAs have been shown to target mRNA of α-synuclein—a protein whose accumulation, aggregation and spread can disrupt neurotransmission and neuronal metabolism compromising neuronal functions already at the early PD stages (26, 30, 31). Mutations in *DICER1* gene and reduced Dicer activity are associated with increased susceptibility to stress, apoptosis, developmental abnormalities, aging, metabolic disorders, disturbed immune system and neuronal functions, and neurodegeneration (29, 32–48). Conversely, increased Dicer levels and/or activity are mostly associated with increased cell survival, but also tumorigenesis (6, 49–52).

The cytoskeleton is an essential cell structure consisting of multiple well-regulated components. It is represented by the three types of filaments: actin filaments, microtubules, and intermediate filaments. These protein complexes form a tight network functioning by assembly-disassembly, which requires not only filaments themselves, but also the number of additional factors. These factors are: nucleation promoting factors for initial filament formation, capping factors preventing filament growth, polymerization factors promoting fast and sustainable filament growth, depolymerization factors for disassembling, crosslinkers and stabilization factors for organizing and building of more complex structures, and adapter proteins to facilitate the multiprotein complex formation on every stage of the process [reviewed in Hohmann and Dehghani (53)].

One of the most interesting aspects for research related to cytoskeleton remodeling is its regulation or changes in regulation during pathophysiological processes, such as neurodegeneration or malignization. During the malignization cell cycle, morphogenesis, and migration ability undergo profound changes mediated by the expression level changes of cytoskeletal additional factors. Transcription factors, nucleation factors, polymerization factors, and certain Rho-GTPases are known to be significantly dysregulated during both initial and promoted carcinogenesis. Also, multiple proteins which are not directly related to cytoskeleton reorganization machinery, but take part in its remodeling as an event, such as transcription factors directly involved in cytoskeleton machinery gene expression or those involved in the epithelial–mesenchymal transition (EMT) are investigated in the context of malignancy (54, 55).

An appropriately organized neuronal cytoskeleton is important for neurodevelopment and normal neuron functioning. Dysregulation of either proper cytoskeletal function or its network proteins (for example capping proteins or those involved in tubulin post-transcriptional regulation) are now the field of interest for new therapeutic targets for neurodegenerative disorders [reviewed in Eira et al. (56)].

As such, the regulation of cytoskeleton dynamics and functions is critical for cell survival. MicroRNAs are known to affect the expression of cytoskeletal proteins both in normal and disease conditions (57–62). We hypothesize that, given the fundamental importance of cytoskeleton dynamics for essential cellular processes, microRNAs regulating it would be involved and affected in neurodegenerative diseases and carcinogenesis—pathological processes which represent two opposite cellular fates: either uncontrolled cell loss or abnormal cell proliferation, migration, and survival. Reviewing the literature on microRNA-dependent regulation of protein expression, we specifically aim to evaluate this hypothesis and find published evidence on whether microRNAs regulating cytoskeleton dynamics, as well as tightly linked clathrin-mediated endocytosis (CME) and cellular migration processes, are critical for both neurodegeneration, and cancer. We suggest that such microRNAs will be very interesting and important subject for more in-depth future studies both from the point of basic biology as well as possible therapeutic agents and/or targets.

## Clathrin-Mediated Endocytosis and Cytoskeleton Dynamics in Neurodegeneration and Cancer

Among all types of cellular transport, CME is currently the best characterized process that has been extensively studied since its discovery in 1964 [reviewed in Maib et al. (63) and Mettlen et al. (64)]. Numerous ligands, including transferrin and epithelial growth factor (EGF), coupled to the corresponding receptors are internalized via CME. The central event of CME is the formation of a 50–100 nm clathrin-coated vesicle (CCV) to transport molecular cargoes inside the cell (65). Generally, the CCV is formed by five consecutive and partially overlapping steps essential for CME [reviewed in McMahon and Boucrot (66)]. The CCV is generated by binding of FCHO1/2 proteins to phosphatidylinositol-4,5-bisphosphate-rich zones at the plasma membrane followed by the formation of the FCHO1/2-EPS15-EPS15R-intersectins complex to recruit AP2 and clathrin for cargo selection and vesicle coating. GTPase dynamin then cuts the CCV from the inner surface of the plasma membrane. When molecular cargo has been delivered, the vesicle is disassembled by ATPase HSC70 recruited by auxilin or cyclin G-associated kinase (GAK). After delivery of molecular cargo and uncoating, the vesicles can be assembled again from free components for the next round of endocytosis.

The complexity of this process can be partially reflected by the quantity of proteins participating at different steps of CCV formation: to date, nearly 60 distinct proteins are known to be involved in CME in yeasts, with 85% of those being homologous to mammalian endocytic proteins (67). Additional proteins can be recruited by main participants, primarily, scaffold and adaptor proteins, such as AP2 and intersectins, at different stages. Altogether, while the main molecular events in CME have been discovered, the detailed mechanisms regulating it are not fully understood.

Being a vital process for cell life and development, CME is tightly linked to multiple cellular pathways and events. For many CME proteins, close association with remodeling and regulation of the cytoskeleton has been demonstrated. By using its DH-domain, a long isoform of mammalian intersectins, adaptor/scaffold protein family participating at the nucleation step of CCV formation interact with guanine-exchange factor Cdc42 regulating its activity in the polymerization of actin cytoskeleton via activation of N-WASP and subsequent formation of filopodia—actin-rich protrusions on the cell surface [reviewed in Herrero-Garcia and O'Bryan (68)]. Recent reports showed the involvement of ITSNs in the formation of mammalian oocytes through the Cdc42 pathway, further confirming their role in the processes of growth and development (69, 70). Additionally to the binding of Cdc42, ITSN1 has been shown to colocalize and physically interact with N-WASP and another cytoskeletal protein, WIP, further promoting filopodia formation (71, 72). Another study reveals the role of ITSN1-L in the actin-dependent dispersal of the Golgi ribbon by interacting with GCC88, the Golgi ribbon modulator (73). Almeida-Souza et al. (74) identified FCHSD2 as the major activator of actin remodeling in CME after recruitment to endocytic pits by ITSN1 via unusual SH3-SH3 interaction. The endocytic protein Numb which is also involved in neural differentiation interacts with Rac and Cdc42 to induce reorganization of actin cytoskeleton and promote cell migration (75). MARK, a microtubule-associated kinase, which post-translationally phosphorylates several microtubule-associated proteins including Tau, is colocalized and copurified with AP2 complex and clathrin, explaining its influence on CCV trafficking (76). Moreover, AP2 controls acetylation of microtubules at clathrin-coated pits via interactions with α-tubulin acetyltransferase αTAT1 thus having impact on the motility of migrating cells (77). Dynamin 2 GTPase, which cuts the CCV from the plasma membrane can enhance cell motility, particularly in cancers, through direct interactions with α-actinin 4 (78) and podocalyxin (79). In myogenesis, dynamin 2 promotes the organization of actin filaments in the invadosome to drive membrane fusion by direct interaction with Tks5, a critical invadosome scaffold protein (80). The role of dynamin as a unique multifilament actin-binding protein was demonstrated in the recently discovered mechanism of dynamin-driven regulation of actin cytoskeleton. This mechanism is based on the ability of dynamin to form an actin-bundling helix with subsequent disassembly facilitating Arp2/3-mediated branched actin polymerization. Dynamin assembly/disassembly cycles promoted continuous actin binding resulting in the formation of mechanically stiff super-bundles of actin (81).

Defects in endocytic machinery are often associated with various disorders and can even be lethal. In neurons, CME is an essential process for the delivery and replenishment of synaptic vesicles carrying neurotransmitters to convert electric stimuli to chemical signals (82–84). Neurodegenerative disorders are linked to abnormalities in CME by mutations in endocytic genes, inappropriate regulation and defective proteins that can abolish uptake of molecular cargoes. In neurons, synaptic vesicle endocytosis is mainly mediated through CME. Mutations in auxilin gene, *DNAJC6*, an important brain-specific protein in CCV uncoating, are associated with juvenile/early onset of PD and development of epilepsy and intellectual disability in addition to typical PD symptoms (85). In *Drosophila* models, downregulation of auxilin leads to the progressive dopaminergic neuronal loss and motor disabilities (86). Phosphorylation of auxilin by LRRK2 results in the accumulation of oxidized dopamine and overexpression of α-synuclein, one of the main pathological proteins in PD development (87). Connor-Robson et al. (88) demonstrated that the G2019S mutation in the *LKKR2* gene in iPSC-derived dopaminergic neurons led to significant deregulation of CME in synaptic vesicles by decreasing levels of endophilin I-III, dynamin 2, and various RAB proteins resulting in a functional impairment of the process. These data provide more evidence of LRRK2-associated disruption of endocytosis in PD development. Mutations in heterotetrameric adaptor complex AP2, which recruits clathrin to the forming pit (89), can lead to the development of various neurodevelopmental and other somatic disorders. As has been shown by Helbig et al. (90), mutations in the μ-subunit-encoding *AP2M1* gene impaired conformational activation and thermodynamic entropy of AP2 complex resulting in the impairment of CME and eventually contributing to the developmental and epileptic encephalopathies.

Another adaptor/scaffold protein, intersectin 1 (ITSN1) has been shown to be deregulated in AD and Down syndrome (DS) patients, as well as in several cancers. In humans, the *ITSN1* gene is located on chromosome 21 and is overexpressed in DS (91). Interestingly, in aged DS patients, levels of ITSN1 are decreased compared to the younger ones. Furthermore, DS patients with concurrent AD diagnosis are characterized by reduced levels of ITSN1 compared to DS-only cases. Mouse models overexpressing the short isoform of ITSN1, ITSN1-S, have reduced locomotor activity and abnormal behavioral phenotype associated with the disruption of neuronal functions due to interference with CME and other signaling pathways (92). ITSN1-S deficiency in acute lung injury contributes to endothelial barrier dysfunction and pulmonary edema (93), whereas in lung cancer it induces ubiquitination of Eps8 oncoprotein leading to the impairment of Rac activation and subsequent decrease of cancer cell migration and metastasis (94). In Huntington's disease, ITSN1 activates c-Jun-NH(2)-terminal kinase (JNK)-MAPK pathway resulting in the increase of aggregate formation by mutant huntingtin (95). Levels of the neuron-specific long isoform of ITSN1, ITSN1-L, are increased in refractory epilepsy patients and rat models (96). Overexpression of ITSN1 leads to anchorage-independent growth and tumorigenesis in neuroblastoma (97, 98) and proliferation of glioblastoma cells through Raf/MEK/ERK pathway activation (99), demonstrating an essential role of ITSN1 in the development of malignancy and tumor progression.

PICALM protein (also known as CALM), which binds the heavy chain of clathrin and thus is involved in clathrin-coated pit assembly (100), plays a role in the development of AD by interacting with tau and mediating endocytosis of α-amyloid protein (APP). In a murine tauopathy model with PICALM haploinsufficiency, Ando et al. (101) demonstrated aggravation of tau pathologies and tau-mediated neurodegeneration, whereas in another study, PICALM overexpression resulted in faster internalization of APP by CME and eventual enhancement of Aβ production (102).

As a complex and multilayered process, CME undergoes regulation at all steps of the formation of CCV by numerous regulatory molecules and mechanisms. For instance, agonist-activated G protein-coupled receptors (GPCRs) are post-translationally phosphorylated and ubiquitinilated leading to the recognition by adaptor proteins β-arrestins and epsin1 and targeting to CCVs (103, 104). The AP2 complex, which is activated by conformational changes in response to phosphatidylinositol-4,5-bisphosphate (PIP2) and binding of molecular cargoes at multiple sites (105), is allosterically regulated by interactions with other adaptor/scaffold proteins involved in CME (106, 107). Binding of AP2 to sorting signals is regulated and enhanced by phosphorylation of its μ2 subunit by AKK (108). Dynamin, another key protein for CME, not only cuts mature pit from the membrane but also regulates CCP maturation (109).

## MicroRNAs Regulating Clathrin-Mediated Endocytosis

Non-coding RNAs, primarily microRNAs, constitute another level of regulation of gene expression, mainly post-transcriptionally. There is still limited information about regulation of intracellular trafficking pathways by microRNAs; however, recent years yielded more evidence of the microRNA-associated regulation of endocytosis. The microRNA cluster miR-17~92 is known as oncomir-1 due to its potent oncogenic function and overexpressed in many cancers. It is a polycistronic cluster that encodes 7 microRNAs which mainly facilitate cell proliferation, malignancy, and tumorigenesis (110–116), although for some microRNAs from this cluster an opposite effect has been shown (117, 118). Known targets of microRNAs encoded by this cluster are largely regulators of cell cycle progression and apoptosis. In addition to these known effects, the role of this cluster in endocytosis has been demonstrated. A member of miR-17~92 cluster, miR-17 regulates endocytic trafficking by targeting TBC1D2/Armus and low-density lipoprotein receptor (LDLR) participating in LDL trafficking. According to Serva et al. (119) miR-17 directly targets 3′UTRs of both genes and downregulates their expression. Consequently, this leads to the reduction of mitotic cell numbers and inhibition of cell proliferation. By regulating endocytic trafficking, miR-17 seed family potentially influences such processes as cell adhesion that, in turn, might cooperate with other functions of miR-17 in health and disease (119).

In gastrointestinal cancers like gastric, colon and liver cancers, microRNAs can regulate not only known cancer-related signaling pathways but also some other processes such as axon guidance, neurotrophin/nerve growth factor signaling, and endocytosis. Zhang et al. (120) confirmed regulation of EGF receptor (EGFR) endocytosis by miR-17 and miR-145, predicted to target endocytosis. While miR-17 promoted, miR-145 inhibited the internalization of EGFR upon EGF binding to the plasma membrane in human colon cancer cells SW1116. Blocking of EGFR endocytosis by miR-145 resulted in prolonged EGFR membrane signaling and altered responsiveness of colon cancer cells to EGFR-targeting drugs (120).

Another microRNA, miR-199, gained particular interest because of its origin from the introns of dynamin (*DNM*) genes. *DNM* genes encode the conserved miR-199a and miR-199b family of miRNAs within their intronic sequences. Sense strands of the *DNM* genes are transcribed and translated to produce DNM proteins that are involved in endosome trafficking. miR-199a-5p is transcribed in the nucleus from the antisense strand of introns in the *DNM2* and *DNM3* genes. miR-199a and miR-199b regulate endocytic transport by downregulating the expression of such endocytic genes as clathrin heavy chain (*CLTC*), Rab5A, *LDLR*, and caveolin-1 (*Cav-1*) leading to the inhibition receptor-mediated endocytosis in human cell lines Huh7 and HeLa (121). Moreover, *DNM2* gene derived miR-199 play roles in ovarian cancer metastasis by the regulation of hypoxia induced factors HIF1 and HIF2. DNM2 levels are reduced in hypoxia due to HIFs that have several binding sites in the *DNM2* promotor region. In epithelial ovarian cancer cells, HIF1α and HIF2α are reciprocally regulated by DNM2. Moreover, miR-199 encoded by the antisense strand of *DNM2*, decreased HIF1α and HIF2α expression leading to the reduction of cell migration and invasion, thus establishing the connection between hypoxia and endocytosis in ovarian cancer (122). In addition to miR-199, two other microRNAs, miR-3120-5p and miR-214-3p, are synthesized from sense and antisense strands of the *DNM3* intron, respectively. Moreover, miR-3120 was demonstrated to be located in neuronal cell bodies and regulate genes of other CME-associated proteins, Hsc70 and auxilin, inhibiting CCV uncoating (123).

ITSNs are involved in multiple processes in the cell. Among them, is regulation of expression of the epithelial sodium channel (ENaC) in the kidney nephron, a major determinant of sodium (Na^+^) and water balance. Aldosterone hormone has been shown to regulate microRNA expression of the mmu-miR-23-24-27 cluster in the cortical collecting duct (CCD) in the kidney nephron both *in vitro* and *in vivo*. In the absence of aldosterone stimulation upregulation of these microRNAs increased Na^+^ transport. ITSN2 has been confirmed as a direct target for miR-27 from the mmu-miR-23-24-27 cluster. Moreover, aldosterone was also able to decrease ITSN2 alongside miR-27. Since ENaC can interact with clathrin with effect on endocytic retrieval from the apical membrane and ITSN2 is involved in facilitation of this event, decrease of ITSN2 levels may delay ENaC removal from the apical surface of CCD (124). Regulation of ITSN1 by microRNAs has also been demonstrated. Lin et al. (125) studied the role of miR-194 in kidneys where it mediates K^+^ intake by the ROMK channel. High K^+^ intake increased mmu-miR-194 levels which in turn led to the increased activity of ROMK channels with the decrease of ITSN1 expression. ITSN1 has been confirmed as a direct target of miR-194 in luciferase assays and Western blot analysis. The authors concluded that miR-194 can regulate ROMK channel activity by modulating ITSN1 levels and possibly enhancing ITSN1/WNK-dependent endocytosis (125).

Perfluorooctanoic acid (PFOA) is used in a vast amount of industrial and consumer products and there are concerns that it may result in testicular toxicity. According to the study performed by Lu et al. (126), PFOA exposure changed levels of the proteins that were involved in endocytosis (and, narrower, receptor-mediated endocytosis) and blood-testis barrier, namely, ITSN1, serine protease inhibitor A3K (Serpina3k), and apolipoprotein a1 (APOA1). In addition to proteins, several microRNAs were also downregulated. Analysis of potential regulatory pairs between deregulated genes and microRNAs after PFOA exposure revealed a set of potential miR-gene pairs, among which a pair of miR-133b-3p/CTLA (clathrin light chain A) was confirmed in the luciferase assay. This suggests a role of miR-133b-3p in the disturbance of receptor-mediated endocytosis following PFOA exposure (126).

## MicroRNAs Regulating Invasiveness and Cell Motility

MicroRNAs play an important role in normal cellular homeostasis via regulating multiple pathways involved in cell growth, proliferation and migration. These cellular activities are dysregulated in cancer cells, leading to uncontrolled tumor growth and metastasis formation. Multiple microRNAs were shown to be significantly up- or downregulated in different cancers. Frequently, their targets are involved in pathways affecting transcription factor repression or activation, chromatin remodeling machinery, cytoskeleton and adhesion molecules, cell cycle regulators, and metabolic genes, for example, involved in glucose metabolism. One microRNA can affect one or multiple targets within one pathway, as well as affect multiple pathways, which makes these molecules promising potential biomarkers and drug targets for tumor treatment and preventing cancer cell migration. The effect of certain microRNA action may differ from cell type to cell type due to different transcriptomes and hence different interactomes, although certain microRNAs may have a constant effect on multiple cell types. In this section, we discuss recent papers demonstrating microRNAs' contribution to neural tumorigenesis with the main focus on glioma as the most aggressive malignancy. Multiple microRNAs regulate cell motility, proliferation and division events targeting different genes, including those involved in cytoskeleton reorganization. For example, members of the microRNA-200 family are involved in regulation of cellular growth and migration via targeting filamin, a protein important for actin filament organization, and RhoGDIA, required for cytoskeleton dynamics (see Table 1) (162, 163). If certain microRNA is dysregulated in specific tumor cells, the rate of dysregulation may be a helpful tool for diagnosis. Certain microRNAs can be potential biomarkers for tumor grade verification, and target identification is important for understanding whether a particular microRNA promotes uncontrolled tumor cell division, or helps to invade, or both, which may help to specify an individual therapeutic strategy. Recent studies show numerous microRNAs to be downregulated in glioma and correlate with tumor grade. For example, expression levels of miR-1254 were significantly lower in glioma cell lines compared to those in normal human astrocytes, as well as in glioma tissue samples in comparison with normal brain tissue. Higher expression level was associated with higher survival rate in patients with primary and recurrent glioma, as well as *in vitro*, upregulated miR-1254 decreased glioma cells invasive, migratory and proliferative potentials via direct targeting of CSF-1, which is known to regulate macrophage motility, migration and maturation (164, 165).

**Table 1:** Role of microRNA regulating some endocytic genes in neurodegeneration and cancer/invasion.

**microRNA**	**Role in endocytosis**	**Role in neurodegeneration**	**Role in cancer and invasion**
miR-17	Regulation of TBC1D2/Armus and LDLR participating in LDL trafficking (119); promotion of EGFR internalization in human colon cancer cells SW1116 (120)	MiR-17 is overexpressed in peripheral mononuclear blood cells (PMBC) from PD patients (127), downregulation of miR-17 led to paraquat-induced dopaminergic degradation (128); miR-17 responses to formaldehyde treatment and modulates EPHA4, GNPDA2 and TXNIP expression in the brains of MGMT–/– mice involved in amyloid deposition, neurotransmission, and response to oxidative stress (129)	miR-17 inhibits HGF/ERBB3-NF-kB positive loop suppressing post-operative metastasis of HCC (130); miR-17 downregulated netrin 4 promoting migration and invasion of breast cancer cells (131); miR-17 targets SIK1 leading to the proliferation and migration of human colorectal cancer cells (132); overexpression of miR-17 in human fibroblasts co-cultured with colon cancer cells remarkably reduced invasion of cancer cells (133); miR-17 targets ETV1 in triple negative breast cancer suppressing proliferation, migration, and tumor metastasis (134)
miR-145	Inhibition of EGFR internalization in human colon cancer cells SW1116 (120)	miR-145-5p downregulated NR4A2 leading to neuronal cell death (135), downregulation of miR-145 induces astrogliosis after spinal cord injury (136), miR-145 is differentially expressed in multiple sclerosis (137)	miR-145 targets CDCA3 leading to the suppression of proliferation, EMT, and metastasis of colorectal cancer (138); in upper tract urothelial carcinoma, miR-145 targets ARF6 repressing cell migration, and invasion (139); miR-145 targets WNT2B leading to suppression of cervical cancer progression and metastasis (140); miR-145 targets NF-kB pathway inhibiting invasion and migration of papillary thyroid carcinoma cells (141); miR-145 targets TWIST leading to the inhibition of migration and invasion of colorectal cancer cells (142)
miR-199	Downregulation of clathrin heavy chain (CLTC), Rab5A, low-density lipoprotein receptor (LDLR), and caveolin-1 (Cav-1) expression leading to the inhibition receptor-mediated endocytosis in human cell lines Huh7 and HeLa (121)	Prenatal circulating miR-199 is upregulated in plasma samples in pregnant women with fetal Down syndrome (143); in CSF of AD patients, miR-199b-5p is expressed more often comparing to other microRNAs (144)	Decrease of HIF1α and HIF2α expression levels leading to the reduction of cell migration and invasion (122); miR-199 targets Snail resulting in inhibition of EMT and invasion of hepatoma cells (145); miR-199 downregulated RGS17 resulting in suppression of proliferation, migration, and invasion of hepatocellular carcinoma cells (146)
miR-3120	Downregulation of Hsc70 and auxilin, inhibiting CCV uncoating (123)	No data (March 2020)	No data (March 2020)
miR-27	Downregulation of ITSN2 resulting in the delay of ENaC removal from CCD in kidneys (124)	No data (March 2020)	Overexpressed miR-27 promoted multiple myeloma cell proliferation, cell migration, and invasion (147); miR-27b targets Sp1 leading to the inhibition of growth and invasion of NSCLC cells (148); miR-27 is overexpressed in invasive adenocarcinomas with the linear increase of microRNA level according to the stage (149); miR-27a targets tumor suppressor MCPH1 in renal cancer (150); upregulation of miR-27a promoted migration, invasion, and angiogenesis of thyroid cancer cells (151)
miR-194	Regulation of ROMK channels activity by downregulation of ITSN1 thus possibly enhancing ITSN1/WNK-dependent endocytosis (125)	In ALS, miR-194 is downregulated (152); in the prefrontal cortex of late-onset AD patients, miR-194 is upregulated (153); in the blood of AD patients, miR-194-5p is significantly downregulated (144)	miR-194 suppresses high glucose-induced progression of non-small cell lung cancer by targeting NFAT5 (154); overexpression of miR-194 in nasopharyngeal carcinoma suppresses cell proliferation, migration, and invasion by targeting MAP3K3 (155); in breast cancer, miR-194-5p promotes cell proliferation, migration, and invasion by targeting SOX17 (156); in prostate cancer, miR-194 is a driver of cancer metastasis promoting migration, invasion, and EMT (157)
miR-133	Decrease of CTLA levels after PFOA exposure leading to the disturbance of receptor-mediated endocytosis (126)	miR-133 is overexpressed in serum of ALS patients (158)	In lung adenocarcinoma metastasis cells, miR-133 acts as a tumor suppressor targeting FLOT2 via Akt signaling pathway (159); in gastric cancer, miR-133 downregulates CDC42 expression, and PAK activation inhibiting cancer cell proliferation and migration (160); in U87 glioma cell lines, miR-133 targets FOXC1 suppressing tumor growth, and invasion (161)

MicroRNAs can both directly affect transcription factors and upstream molecules in the same signaling pathway. Upregulation of miR-129-5p in glioblastoma cell lines LN229 and A172 led to significant reduction of cell proliferation and colony formation ability, also wound closure potential of the cells overexpressing mir-129-5p was lower compared to intact cells. ZFP36L1, an early response gene, was identified as a downstream target of miR-129-5p, promoting cell invasiveness, proliferation and migration (166). *In vitro*, overexpressed miR-1471 showed less proliferation activity compared to the control group, moreover, ectopic expression of miR-1471 could inhibit glioma cell line invasion ability. Such effects were caused by direct targeting of metadherine, which has dual roles in metastasis seeding and promoting chemoresistance in breast cancer (167, 168). Interestingly, MTDH is required for RISC complex formation and its optimal activity, which can affect miR-induced silencing by miR-1471, and it also interacts with spliceosome proteins YTHDC1, Sam68, and T-STAR, thus playing an important role in alternative splicing events (169, 170).

miR-940 was also significantly downregulated in glioma samples and overexpression in cell lines U87 and LN229 dramatically decreased expression levels of mesenchymal markers, such as N-cadherin, Vimentin, Fibronectin, α-SMA, and MMP2. On the other hand, epithelial marker E-cadherin was upregulated compared to the control group. Migration and invasion rates of glioma cells overexpressing miR-940 were significantly reduced compared to control groups, and it was shown that miR-940 influences these cell abilities through the EMT pathway by targeting ZEB2—a transcription factor activated by TGFβ (171). Also, miR-940 can induce G1/S phase arrest and increase apoptosis rate via targeting MTHFD, an enzyme participating in one-carbon metabolism of folate in mitochondria, which makes miR-940 a promising biomarker and therapeutic target for glioma (172, 173). Another zinc finger protein, ZBTB20, was upregulated in GBM samples. It is involved in glucose and lipid metabolism and is also associated with the progression of hepatocellular carcinoma as an independent marker (174–176). It was significantly upregulated in GBM cells but overexpression of miR-758-5p resulted in decreasing ZBTB20 expression levels and inhibition of invasion, proliferation and migration of GBM cells (177).

The most important features of invasive cells are their ability to lose adhesion, anchorage-independent growth and capacity to degrade extracellular matrix which helps to reach not only adjusted tissues, but also distinct organs. Such activities require a large number of different proteins involved in cytoskeleton reorganization, matrix degradation and signaling, thus multiple papers investigate microRNAs as regulators of invasion-related genes.

Genome-wide analysis of potential miR-200 targets performed by Bracken's group revealed that this microRNA can regulate invadopodia and focal adhesion through multiple signaling, for example inhibiting Rho signaling either via its activators, ARHGEF3 and NET1, and multiple downstream effectors, and effectors of effectors. It may also affect the number of transcription factors involved in invasion such as ZEB1, SUZ12, STAT5B, E2F3, TCF12, CTNNB1, and several SMADs (178). The fibulin glycoproteins family functions as regulators of cell morphology, adhesion, proliferation and motility. Fibulin-3 was shown to regulate matrix metaloproteinases (MMPs) and tissue inhibitors of MMPs. It can either promote or repress malignant behavior due to different cell context, but in glioma cells it is upregulated, increasing invasion rates. miR-338-5p directly targets its 3′UTR and serves as a tumor suppressor in glioma (179). Moreover, miR-338-5p may regulate FOXD1, which leads to suppression of MAPK-signaling in glioma cells and therefore less proliferation and invasion activity (180). miR-93-5p could repress invasion by direct targeting of MMP2, which is essential for ECM digestion and subsequent migration of tumor cells (181). Interestingly, miR-10b regulated the orchestra of transcription factors such as HOXD10, PAX6, TP53, and NOTCH1, repressing invasion-related genes RHOC, PLAUR, MMP2, and CTNNB1, and decreasing the invasive potential of mesenchymal type glioma cell line U87-2M1 (182). MicroRNA-145 was significantly downregulated in glioma tissues, as well as in U87, and U251 cell lines. ROCK1, its target, is one of the key players of actin cytoskeleton reorganization, which promotes tumor cell invasion through affecting the RhoA/ROCK1 signaling pathway (183). Interestingly, certain microRNAs may be potentially used as the differential markers for glioma grade diagnostics due to the changes of their expression level during tumor progression, making microRNA profiling of different tumors at different stages a very promising tool for further understanding of malignancy mechanisms (184).

## Common microRNAs in Cancer and Neurodegeneration

As discussed above, the processes of CME, invasiveness, and cell motility are intrinsically linked by the cytoskeleton and its dynamic reorganization. MicroRNAs regulating genes involved in cytoskeleton dynamics can modulate cell invasiveness, motility, and CME and thus also neuronal physiology and survival. Since the general mechanism of CME is universal for all cells and tissues it can be hypothesized that endocytic genes may undergo similar regulation in neurons as they are regulated in non-neuronal cells. While further studies addressing the role of microRNAs in post-mitotic neurons are clearly necessary, can we attempt to extrapolate the findings from cultured cell lines, if particular microRNAs and their targets are expressed in neurons?

Starting from the hypothesis about the universal mechanism of post-transcriptional regulation of the cytoskeleton and CME proteins, we reviewed here the literature describing the role of cytoskeleton- and endocytic-associated microRNAs in neurodegeneration and cancer/invasion, aiming to find possible common microRNAs. However, according to the current literature, there is no significant overlap in microRNAs in neurodegeneration and cancer. Some microRNAs associated with endocytosis participate both in neurodegeneration and tumorigenesis (Table 1). However, the same microRNA molecules are reported to target different genes in different cellular contexts. It is, therefore, important to be careful when making profound conclusions about “universal” microRNAs regulating all processes in the same manner in different cell types, for example, when extrapolating findings from cultured cells to post-mitotic neurons. On the other hand, the absence of clear evidence about CME regulation in neurons by microRNAs regulating endocytosis in non-neuronal cells does not necessary imply the absence of the regulation itself. Compared to immortalized cancer cells, neurons are technically challenging to work with, as they are not dividing, difficult to transfect and culture, and, accordingly, only a handful of studies directly addresses microRNA functions in neuronal cells. Moreover, post-transcriptional regulation of some isoforms may be restricted to neurons or even particular types of them complicating studies of gene regulation in neurons even more. Multiple evidences demonstrate the presence of neuron-specific transcripts as well as specific regulation of widely expressed genes. A long isoform of human ITSN1 gene, ITSN1-L, which is expressed primarily in neurons carries unusually long 3′UTR of 11,561 nucleotides suggesting neuronal-specific regulation of this transcript although no data supports this hypothesis so far (185). Two isoforms with different 3′UTRs of the major cytoskeletal protein β-actin coexist in mouse neurons with much lower expression level, but significantly higher translational efficiency of the transcript with longer 3′UTR. Interestingly, the β-actin transcript with longer 3′UTR harbors conservative target site of mmu-miR-34, which upregulates β-actin expression (186). Brain-derived neurotrophic factor (BDNF) critical for the development and plasticity of neurons undergoes transcript-specific regulation by miR-206 inhibiting only an isoform with long 3′UTR without affecting a transcript with a shorter 3′UTR. Such specific regulation led to the decrease of BDNF expression in cell bodies and axons (187). Another example of transcript-specific 3′UTR with distinct regulation is Nurr1 transcription factor. Its long 3′UTR is targeted by miR-93, miR-204, and miR-302d in rat mesencephalic neurons resulting in fine-tuning of Nurr1 transcript-specific expression and possibly affecting the development and survival of dopaminergic neurons (188). Single nucleotide polymorphisms (SNPs) and short structural variants (SSVs) may also affect microRNA binding sites and mRNA expression. However, in contrast to long splice- and transcript-specific variants of 3′UTRs the evidence of such regulation is limited. Genetic variations of α-synuclein 3′UTR were found in PD and Dementia with Lewy Bodies (DLB) patients, however, no specific microRNAs binding these sites were identified (189, 190).

Existence of tissue-specific transcripts can partially explain difficulties in the understanding of microRNA-driven regulation of a particular cellular pathway. Thus, for any particular microRNA, the effort should be made to identify the majority of its targets in different cell types, which can be done by profiling mRNAs after overexpression or inhibition of a particular microRNA. With current advances in RNA sequencing it becomes increasingly possible. Only when we have a more complete picture of the scope of particular microRNA targets, we can make clear conclusions about whether these are the same or different in different cell types.

With this review of the current literature, we argue that more research specifically focused on the role of microRNAs regulating CME and cytoskeleton dynamics in neurons is urgently needed. Currently, we can conclude that at least some endocytic-associated microRNAs with strictly proven roles in cancer and neurodegeneration are worth paying more attention to as possible therapeutic targets.

## Future Perspectives

Multiple studies discussed above indicate the important role of microRNAs in regulation of CME and cell invasiveness. Nevertheless, many publications rely on studies in cultured cells, which is especially problematic when such results are extrapolated to post-mitotic neurons. The results obtained using reporter constructs to demonstrate the targeting of a particular gene with a selected microRNA must be confirmed by demonstrating its effect on an endogenous target. Frequently, non-physiological levels of synthetic microRNA mimics and/or inhibitors are transfected to cells, even though their unspecific effects are well-known (191). Further, as one microRNA is capable of regulating multiple targets, the effects of microRNA mimics and inhibitors will affect all targets of this particular microRNA, and therefore, the results of such experiments must be interpreted with caution. This problem can be overcome by the use of specific target protectors, disrupting the interaction of particular microRNA with its selected mRNA target (192). When uncovering microRNA regulatory networks and targets, we must aim at demonstrating that endogenous microRNA can regulate the endogenous target mRNA in the selected cell type of interest resulting in measurable functional effects. We should also bear in mind species-specific differences in microRNAs as well as in 3′UTR sequences and, therefore, in microRNA regulatory networks, which has already been demonstrated at least for some genes (193, 194). Also, the further advance of methods to study microRNA/mRNA interactions (195) as well as bioinformatic algorithms for microRNA target prediction (196) will improve our understanding of microRNA regulatory networks in different cellular processes. Clearly, more careful and controlled research is needed to address the importance and the extent of microRNA-mediated regulation of CME and cell motility. This will hopefully lead to discovery of new targets and the development of microRNA-based therapies for cancer and neurodegeneration.

## Author Contributions

DG, AH, and AD: conceptualization, writing, original draft preparation, writing, review, and editing. AD: supervision. DG and AD: funding acquisition. All authors contributed to the article and approved the submitted version.

## Conflict of Interest

The authors declare that the research was conducted in the absence of any commercial or financial relationships that could be construed as a potential conflict of interest.
